# Environmental Considerations in Neurodegenerative Disease Etiology and Diagnosis

**DOI:** 10.1002/alz70856_102705

**Published:** 2025-12-24

**Authors:** Hassan A. N. El‐Fawal

**Affiliations:** ^1^ Institute of Global Health and Human Ecology, American University in Cairo, Cairo, Egypt

## Abstract

Given the idiopathic nature of many neurodegenerative diseases (ND), the contributions of environmental factors need to be considered. Here environment is defined as the milieu of chemicals, whether occupational and/or ecological exposures, rural and urban spheres, as well as social stressors. This is particularly of concern in absence of enforced regulatory oversight. It should not be assumed that this is an issue exclusive to developing countries, given concerns of environmental equity globally. Over the past 3 decades, numerous studies have implicated gene‐environmental interactions as playing a role in precipitating neurotoxicity and neurodegenerative conditions. Indeed, several studies have drawn attention to the potential contribution of the environment, including fetal, to cognitive decline and neurological dysfunction. A pilot study of patients with MCI (*n* = 20) as determined by MMSE (<25) and a socioeconomically matched control group (*n* = 30). Sera were analyzed for the presence of neuroantibodies (NAb), IgM and IgG, against neurofilament triplet (NF), glial fibrillary acidic protein (GFAP), and myelin basic protein (MBP) by ELISA, as well as levels of heavy metals (μg/dl) Cd, Pb, Cu, Mn and Al by ICP. There was a go highly significant (*p* = 0.00001) prevalence of Nab against NFL, IgM and IgG, as well as NFH and GFAP, IgG in MCI patients. Significant odds ratios were 23 (95% CI= 1.358 to 422), 8 (95% CI= 1.492 to 21.418), and 33 (95% CI=2.2906 to 146.5430), respectively, for NFL, IgM and NFH IgG. Stepwise multirgeression analysis indicated that urban environment, Al, and MCI diagnosis were significant determinants of Anti‐NFL titers (R^2^=0.6; *p* = 0.00001; 95% CI=‐0.112‐2.60694), whereas diagnosis and Cr where determinants of Anti‐NFH IgG (R^2^=0.37; *p* = 0.00001; 95% CI=‐0.84799‐1.83). In contrast Anti‐GFAP IgG titers were significantly associated with Pb, Mn and MMSE (R^2^=0.47; *p* = 0.00001; 95% CI= ‐0.006‐4.7). In turn MMSE was associated with urban environment, Pb, anti‐NFM and anti‐GFAP titers (R2=0.47; *p* = 0.00001; 95% CI= 10‐16). This preliminary study with the strong association between serum NAb titers and environmental metals, as well as the association of clinical diagnosis with environment, metals and NAb underscores the need to consider these and other environmental factors in dementia, as well as other ND, as will be illustrated.

